# Risk of a Second Primary Cancer after Non-melanoma Skin Cancer in White Men and Women: A Prospective Cohort Study

**DOI:** 10.1371/journal.pmed.1001433

**Published:** 2013-04-23

**Authors:** Fengju Song, Abrar A. Qureshi, Edward L. Giovannucci, Charlie S. Fuchs, Wendy Y. Chen, Meir J. Stampfer, Jiali Han

**Affiliations:** 1 Department of Epidemiology, Tianjin Medical University Cancer Institute and Hospital, Tianjin, China; 2 Department of Dermatology, Brigham and Women's Hospital, Harvard Medical School, Boston, Massachusetts, United States of America; 3 Channing Division of Network Medicine, Department of Medicine, Brigham and Women's Hospital and Harvard Medical School, Boston, Massachusetts, United States of America; 4 Department of Epidemiology, Harvard School of Public Health, Boston, Massachusetts, United States of America; 5 Department of Nutrition, Harvard School of Public Health, Boston, Massachusetts, United States of America; 6 Department of Medical Oncology, Dana Farber Cancer Institute, Boston, Massachusetts, United States of America; McGill University, Canada

## Abstract

**Background:**

Previous studies suggest a positive association between history of non-melanoma skin cancer (NMSC) and risk of subsequent cancer at other sites. The purpose of this study is to prospectively examine the risk of primary cancer according to personal history of NMSC.

**Methods and Findings:**

In two large US cohorts, the Health Professionals Follow-up Study (HPFS) and the Nurses' Health Study (NHS), we prospectively investigated this association in self-identified white men and women. In the HPFS, we followed 46,237 men from June 1986 to June 2008 (833,496 person-years). In the NHS, we followed 107,339 women from June 1984 to June 2008 (2,116,178 person-years). We documented 29,447 incident cancer cases other than NMSC. Cox proportional hazard models were used to calculate relative risks (RRs) and 95% confidence intervals (CIs). A personal history of NMSC was significantly associated with a higher risk of other primary cancers excluding melanoma in men (RR = 1.11; 95% CI 1.05–1.18), and in women (RR = 1.20; 95% CI 1.15–1.25). Age-standardized absolute risk (AR) was 176 in men and 182 in women per 100,000 person-years. For individual cancer sites, after the Bonferroni correction for multiple comparisons (*n* = 28), in men, a personal history of NMSC was significantly associated with an increased risk of melanoma (RR = 1.99, AR = 116 per 100,000 person-years). In women, a personal history of NMSC was significantly associated with an increased risk of breast (RR = 1.19, AR = 87 per 100,000 person-years), lung (RR = 1.32, AR = 22 per 100,000 person-years), and melanoma (RR = 2.58, AR = 79 per 100,000 person-years).

**Conclusion:**

This prospective study found a modestly increased risk of subsequent malignancies among individuals with a history of NMSC, specifically breast and lung cancer in women and melanoma in both men and women.

*Please see later in the article for the Editors' Summary*

## Abstract 2

Studies have suggested a positive association between history of non-melanoma skin cancer (NMSC) and risk of subsequent cancer at other sites. This prospective study found a modestly increased risk of subsequent malignancies among individuals with a history of NMSC, specifically breast and lung cancer in women and melanoma in both men and women.

## Editors' Summary

**Background:**

In the United Kingdom and the United States, about one in three people develop cancer during their lifetime and, worldwide, cancer is responsible for 13% of all deaths. Primary cancer, which can develop anywhere in the body, occurs when a cell begins to divide uncontrollably because of alterations (mutations) in its genes. Additional mutations allow the malignancy to spread around the body (metastasize) and form secondary cancers. The mutations that initiate cancer can be triggered by exposure to carcinogens such as cigarette smoke (lung cancer) or the ultraviolet (UV) radiation in sunlight (skin cancers). Other risk factors for the development of cancer include an unhealthy diet, physical inactivity, and alcohol use. In the United States, the most common cancer is non-melanoma skin cancer (NMSC). Although more than 2 million new cases of NMSC occur each year, fewer than 1,000 people die annually in the United States from the condition because the two types of NMSC—basal cell carcinoma and squamous cell carcinoma—rarely metastasize and can usually be treated by surgically removing the tumor.

**Why Was This Study Done?:**

Some studies have suggested that people who have had NMSC have a higher risk of developing primary cancer at other sites than people who have not had NMSC. Such a situation could arise if exposure to certain carcinogens initiates both NMSC and other cancers or if NMSC shares a molecular mechanism with other cancers such as a deficiency in the DNA repair mechanisms that normally remove mutations. If people with a history of NMSC are at a greater risk of developing further cancers, a specific surveillance program for such people might help to catch subsequent cancers early when they can be successfully treated. In this prospective cohort study, the researchers examine the risk of primary cancer according to personal history of NMSC in two large US cohorts (groups)—the Health Professionals Follow-up Study (HPFS) and the Nurses' Health Study (NHS). The HPFS, which enrolled 51,529 male health professionals in 1986, and the NHS, which enrolled 121,700 female nurses in 1976, were both designed to investigate associations between nutritional factors and the incidence of serious illnesses. Study participants completed a baseline questionnaire about their lifestyle, diet and medical history. This information is updated biennially through follow-up questionnaires.

**What Did the Researchers Do and Find?:**

The researchers identified 36,102 new cases of NMSC and 29,447 new cases of other primary cancers from 1984 in white NHS participants and from 1986 in white HPFS participants through 2008. They then used statistical models to investigate whether a personal history of NMSC was associated with a higher risk of subsequent primary cancers after accounting for other factors (confounders) that might affect cancer risk. A history of NMSC was significantly associated with an 11% higher risk of other primary cancers excluding melanoma (another type of skin cancer that, like NMSC, is linked to overexposure to UV light) in men and a 20% higher risk of other primary cancers excluding melanoma in women; a significant association is one that is unlikely to have happened by chance. The absolute risk of a primary cancer among men and women with a history of NMSC was 176 and 182 per 100,000 person-years, respectively. For individual cancer sites, after correction for multiple comparisons (when several conditions are compared in groups of people, statistically significant differences between the groups can occur by chance), a history of NMSC was significantly associated with an increased risk of breast and lung cancer in women and of melanoma in men and women.

**What Do These Findings Mean?:**

These findings suggest that there is a modestly increased risk of subsequent malignancies among white individuals with a history of NMSC. Although the researchers adjusted for many confounding lifestyle factors, the observed association between NMSC and subsequent primary cancers may nevertheless be the result of residual confounding, so it is still difficult to be sure that there is a real biological association (due to, for example, a deficiency in DNA repair) between NMSC and subsequent primary cancers. Because of this and other study limitations, the findings reported here should be interpreted cautiously and do not suggest that individuals who have had NMSC should undergo increased cancer surveillance. These findings do, however, support the need for continued investigation of the apparent relationship between NMSC and subsequent cancers.

**Additional Information:**

Please access these Web sites via the online version of this summary at http://dx.doi.org/10.1371/journal.pmed.1001433.

- The US National Cancer Institute provides information on all aspects of cancer and has detailed information about non-melanoma skin cancer for patients and professionals (in English and Spanish)
- The non-profit organization American Cancer Society provides information on cancer and how it develops and specific information on skin cancer (in several languages); its website includes personal stories about cancer
- The UK National Health Service Choices website includes an introduction to cancer and a page on non-melanoma skin cancer
- The non-profit organization Cancer Research UK provides basic information about cancer and detailed information on non-melanoma skin cancer

## Introduction

Non-melanoma skin cancer (NMSC) is the most common cancer in the United States. It consists mainly of basal cell carcinoma (BCC) and squamous cell carcinoma (SCC). Its incidence has been rapidly increasing over the past several decades and the incidence rate was about 6,000/100,000 in the year 2006 [1]. NMSC has a low mortality rate of 1/100,000 [2], but its high prevalence and the expense of related treatment make NMSC a major public health problem and place it among the costliest cancers in the United States [3]. Individuals with personal history of NMSC may be at an altered risk for developing other primary cancers [4]–[11]. One view is that sunlight causes NMSC but also produces vitamin D, which in turn may reduce the risk of other cancers [12]. Another view is that NMSC and other cancers may share common carcinogenic exposures or molecular mechanisms in their etiology, such as DNA repair deficiency and immune suppression, and thus the history of NMSC may indicate an increased risk of subsequent cancer development.

Previous studies suggest a positive association between personal history of NMSC and risk of subsequent cancer at other sites [4]–[11]. Most previous reports, however, were based on cancer registry data without adjustment for potential confounding lifestyle factors [4]–[10]. The only cohort study was limited by its sample size and lacked adequate power to assess individual cancer sites [11]. We carried out a cohort analysis to evaluate the association between personal history of NMSC and subsequent malignancy in the Nurses' Health Study (NHS) and the Health Professionals' Follow-up Study (HPFS).

## Methods

### Ethics Statement

Our study was approved by the Human Research Committee at the Brigham and Women's Hospital with written informed consent from all participants.

### Study Population

The NHS was established in 1976, when 121,700 registered nurses aged 30–55 y in 11 US states responded to a baseline questionnaire regarding risk factors for cancer. Participants completed self-administered, mailed follow-up questionnaires biennially with updated information on their lifestyle, diet, and medical history. The HPFS began in 1986 when 51,529 US male health professionals, including dentists, veterinarians, pharmacists, and optometrists aged 40–75 y, completed a baseline questionnaire on lifestyle, diet, and newly diagnosed diseases. The information was updated biennially with follow-up questionnaires. The follow-up rates of the participants in both cohorts exceed 90%. These studies were approved by the Human Research Committee at Brigham and Women's Hospital. Race was self-identified in this study as White, Asian, African American, and others. Only white participants were included in this study, accounting for 95.6% of the total population in the two cohorts. The rationale for focusing the primary hypothesis on white participants only was that the patterns of incidence (and likely the risk factors) for NMSC differ widely by race.

### Identification of NMSC and Other Primary Cancers

We have routinely identified cases of NMSC and other primary cancers in both cohorts (from 1984 in the NHS and from 1986 in the HPFS). Participants reported new diagnoses biennially. With their permission, participants' medical records were obtained and reviewed by physicians to confirm their self-reported diagnosis. Medical records were not obtained for self-reported cases of BCC, because the validity of BCC self-reports was more than 90% in validation studies in our cohorts in early years [13],[14]. The personal history of pathologically confirmed invasive SCC and self-reported BCC was the exposure in this analysis. The study outcome was the occurrence of the first confirmed primary cancer other than NMSC. All other cancer cases were documented by medical records or death certificates, and only confirmed cases were included in the analysis.

### Assessment of Covariates

Covariates in this analysis included age (continuous variable), body mass index (BMI) (<21, 21–23, 23–25, 25–27, 27–29, 29–31, >31), physical activity (quintiles), smoking status (never, past 1–14 cigarettes per day, past 15+ cigarettes per day, current 1–14 cigarettes per day, current 15+ cigarettes per day), multi-vitamin use (yes or no), menopause status and hormone replacement therapy use in women (pre-menopause, post-menopause non-user, post-menopause past user, and post-menopause current user), and physical examination in the last 2 y (yes or no). We asked about the location of residence (US states) at birth and at age of 15 and 30. The 50 states (and the District of Columbia) were divided into three ultraviolet (UV) index groups: 5 or less (low UV index); 6 (medium UV index); and 7 or more (high UV index) [15]. We defined participants in these three groups if they resided in the same UV-index region at birth, age of 15 and 30.

### Statistical Analysis

Follow-up began in 1984 for the NHS and 1986 for the HPFS when the diagnosis of NMSC was first routinely collected, and follow-up ended in 2008 for both cohorts. Participants who reported a history of cancer (including NMSC) prior to baseline were excluded. Participants contributed person-time from the date of return of the baseline questionnaire (1984 in NHS and 1986 in HPFS) until date of diagnosis of confirmed primary cancer, date of death, or the end of follow-up (May 31, 2008), whichever came first. For those who were lost to follow-up, we censored them at the return date of the last questionnaire. Cox regression analysis with time-dependent covariates was used to determine the relative risks (RRs) and 95% CIs of second primary malignancies associated with a previous NMSC diagnosis. We calculated age-standardized absolute risks (ARs) of second primary malignancies associated with a previous NMSC diagnosis. NMSC diagnosis could change during the follow-up period. For individuals with no personal history of cancer at baseline who went on to be diagnosed with NMSC as a first cancer diagnosis during follow-up, the follow-up period before the NMSC diagnosis contributed person-time to the non-exposure group, and the follow-up period after the NMSC diagnosis contributed person-time to the exposure group. Age was coded as a continuous variable in all the analyses. We showed overall cancer risk with and without melanoma. We performed several secondary analyses. We excluded those diagnosed with other primary cancers within the first 4 y of NMSC diagnosis to minimize the detection bias. We examined BCC and SCC history separately. We performed stratified analysis according to age (≤60 y, >60 y), UV-index of residence at birth, age 15, and age 30 (≤5,  = 6, ≥7), smoking status (never smoker, past smoker, current smoker), and BMI (<25, 25–30, ≥30). We coded these factors as dummy variables and tested their interactions with the history of NMSC individually. We tested multiplicative interaction terms by the likelihood ratio test comparing the model with the cross-product terms with the model containing just the main effects of these factors and the history of NMSC along with the same covariates.

We assessed the association between NMSC diagnosis and risk of developing site-specific cancers that were diagnosed in more than 100 patients in each cohort. For individual cancer sites, the Cox models additionally included risk factors specific for some cancer sites. We included additional covariates in the multivariate models for breast, ovarian, endometrial, prostate cancers, and melanoma. The Bonferroni correction for *p*-value was applied for multiple comparisons for individual cancer sites among men and women, calculated as 0.05/n (*n* = 28). Statistical analyses were conducted using SAS software (version 9, SAS Institute). All statistical tests were two-sided.

## Results

Characteristics of our study population according to a personal history of NMSC in mid-point of the follow-up (1998) are shown in Table 1. Participants with a history of NMSC were more likely to be older and tended to burn and have more severe sunburns. Participants with history of NMSC diagnosis were more likely to have red or blonde hair and to reside in high UV-index states. Other characteristics were similar between the exposure group and the non-exposure group.

**Table 1:** Characteristics according to personal history of non-melanoma skin cancer in 1998.

Characteristics^a^	Men (HPFS)		Women (NHS)	
	No NMSC (*n* = 33,995)	NMSC (*n* = 4,561)	No NMSC (*n* = 83,765)	NMSC (*n* = 11,068)
Age, y (SD)	63.9 (9.2)	67.3 (9.3)	63.5 (7.1)	66.4 (6.8)
BMI, kg/m^2^ (SD)	26.2 (3.8)	26.0 (3.7)	26.7 (5.4)	26.1 (5.0)
Physical activity, met-h/wk (SD)	34.6 (39.8)	36.4 (38.3)	17.1 (21.3)	18.3 (21.3)
Tendency to get painful sunburn, %	21	29	13	20
Severe sunburns >5, %	35	44	7	12
Red or blonde hair, %	14	17	15	21
UV-index at birth, age 15, and age 30 ≥7, %	27	32	8	13
Current smoker, %	6	5	11	10
Multi-vitamin user, %	51	57	62	65
Physical examination in the last 2 y, %	84	87	92	93
Red meat, servings/day (SD)	1.2 (0.9)	1.1 (0.8)	0.8 (0.4)	0.7 (0.4)
Fruit and vegetable, servings/day (SD)	3.3 (1.6)	3.3 (1.5)	3.2 (1.4)	3.2 (1.4)
Alcohol, gram/day (SD)	11.0 (14.1)	11.8 (14.7)	4.9 (9.0)	5.6 (9.5)

a Variables were age-standardized except for age.

SD, standard deviation.

We followed the HPFS participants from 1986 to 2008, for a total of 833,496 person-years. During this period, 1,577 cases of SCC, 10,422 cases of BCC, and 10,590 primary cancer cases other than NMSC were recorded. The mean time for the development of a primary cancer after NMSC was 116±47 mo. We followed the NHS from 1984 to 2008 (2,116,178 person-years) during which 2,322 cases of SCC, 21,781 cases of BCC, and 18,857 primary cancer cases other than NMSC were recorded. The mean time for the development of a primary cancer after NMSC was 156±71 mo.

A personal history of NMSC was associated with a higher risk of other primary cancers in men (RR = 1.15; 95% CI 1.09–1.22, *p*<0.0001) and women (RR = 1.26; 95% CI 1.21–1.31, *p*<0.0001) (Table 2). The association attenuated slightly when melanoma was excluded from the outcome in the analysis, but remained significant in men (RR = 1.11; 95% CI 1.05–1.18, *p* = 0.0007) and in women (RR = 1.20; 95% CI 1.15–1.25, *p*<0.0001). Age-standardized AR was 176 in men and 182 in women per 100,000 person-years. The association remained significant after we excluded those diagnosed with other primary cancers within the first 4 y of NMSC diagnosis in men (RR = 1.15; 95% CI 1.05–1.25) and women (RR = 1.19; 95% CI 1.11–1.28). In men, the association was significant according to BCC diagnosis (RR = 1.17; 95% CI 1.10–1.24) but not SCC diagnosis (RR = 1.01; 95% CI 0.87–1.17). In women, the association was significant for both BCC (RR = 1.25; 95% CI 1.20–1.30) and SCC diagnosis (RR = 1.24; 95% CI 1.10–1.39). We compared people with personal history of SCC with people with personal history of BCC on their risk of developing subsequent cancer, and no significant differences were found (Table S1). In addition, we have compared SCC in situ group with invasive SCC group and the group of SCC or BCC; the results are shown in Table S2. Compared to those with history of invasive SCC or those with history of SCC or BCC, individuals with history of SCC in situ were less likely to develop subsequent cancers. Such risk reduction was significant among women.

**Table 2:** Overall and stratified analysis of risks of total subsequent primary cancers according to personal history of non-melanoma skin cancer in men and women.

Group	Cases/Person-Years		AR	Age-Adjusted RR (95% CI)^a^	Multivariable-Adjusted RR (95% CI)^b^
	No NMSC	NMSC			
**Men (HPFS)**					
Overall	9,068/756,608	1,522/76,888	286	1.17 (1.11–1.24)	1.15 (1.09–1.22)
Overall excluding melanoma	8,513/749,343	1,380/75,916	176	1.13 (1.06–1.19)	1.11 (1.05–1.18)
SCC diagnosis history	10,405/824,054	185/9,442	110	1.04 (0.89–1.20)	1.01 (0.87–1.17)
BCC diagnosis history	9,253/766,050	1,337/67,446	305	1.18 (1.12–1.26)	1.17 (1.10–1.24)
Stratified analysis					
Age ≤60 y	1,951/380,536	164/18,175	220	1.26 (1.07–1.48)	1.24 (1.05–1.45)
Age >60 y	7,117/376,072	1,358/58,713	292	1.16 (1.09–1.23)	1.14 (1.07–1.21)
UV-index ≤5	1,181/91,561	204/9,081	370	1.24 (1.05–1.46)	1.23 (1.04–1.46)
UV-index = 6	2,498/196,219	451/20,502	402	1.15 (1.03–1.28)	1.15 (1.03–1.28)
UV-index ≥7	1,232/109,597	252/12,589	362	1.23 (1.06–1.43)	1.24 (1.07–1.44)
Never-smoker^c^	3,042/292,695	538/28,361	357	1.19 (1.08–1.31)	1.19 (1.08–1.31)
Past-smoker^c^	4,049/292,912	707/32,944	262	1.12 (1.03–1.22)	1.12 (1.03–1.22)
Current-smoker^c^	645/46,144	72/2,962	350	1.01 (0.73–1.40)	1.05 (0.75–1.46)
BMI<25	3,890/315,048	660/33,540	258	1.15 (1.06– 1.26)	1.14 (1.05–1.25)
25≤BMI<30	4,152/354,538	693/34,967	321	1.22 (1.12–1.33)	1.20 (1.10–1.31)
BMI≥30	1,026/87,023	169/8,381	251	1.13 (0.94–1.36)	1.11 (0.92–1.34)
**Women (NHS)**					
Overall	16,068/1,900,258	2,789/215,920	254	1.29 (1.23–1.34)	1.26 (1.21–1.31)
Overall excluding melanoma	15,286/1,887,665	2,501/213,349	182	1.22 (1.17–1.27)	1.20 (1.15–1.25)
SCC diagnosis history	18,575/2,095,770	282/20,408	320	1.28 (1.13–1.44)	1.24 (1.10–1.39)
BCC diagnosis history	16,350/1,920,665	2,507/195,512	246	1.27 (1.22–1.33)	1.25 (1.20–1.30)
Stratified analysis					
Age ≤60	5,435/928,744	422/49,999	165	1.31 (1.18–1.44)	1.28 (1.16–1.42)
Age >60	10,633/937,361	2,367/160,661	298	1.28 (1.22–1.34)	1.25 (1.20–1.31)
UV-index ≤5	2,641/318,533	376/31,218	186	1.14 (1.02–1.27)	1.14 (1.02–1.27)
UV-index = 6	5,230/608,658	987/73,647	258	1.26 (1.18–1.36)	1.28 (1.19–1.37)
UV-index ≥7	735/86,789	188/15,431	172	1.16 (0.98–1.38)	1.17 (0.98–1.40)
Never-smoker^c^	6,046/825,740	1,011/92,124	228	1.30 (1.22–1.39)	1.28 (1.20–1.38)
Past-smoker^c^	6,840/742,002	1,399/100,075	258	1.28 (1.20–1.35)	1.27 (1.20–1.35)
Current-smoker^c^	2,697/253,209	346/21,941	195	1.10 (0.98–1.24)	1.10 (0.97–1.23)
BMI<25	7,177/922,638	1,422/111,181	291	1.37 (1.37–1.46)	1.31 (1.23–1.39)
25≤BMI<30	5,294/600,278	871/68,370	216	1.23 (1.14–1.32)	1.21 (1.12–1.30)
BMI≥30	3,597/377,342	496/36,369	242	1.23 (1.12–1.36)	1.23 (1.12–1.36)

a RR adjusted for age (continuous variable).

b Multivariate RR adjusted for age (continuous variable), BMI (<21, 21–23, 23–25, 25–27, 27–29, 29–31, >31), physical activity (quintiles), smoking status (never, past 1–14 cigarettes per day, past 15+ cigarettes per day, current 1–14 cigarettes per day, current 15+ cigarettes per day), multi-vitamin use (yes or no), UV-index of residence at birth, age 15, and age 30 (≤5, 6, ≥7), physical examination in the last 2 y (yes or no), and menopause status and hormone replacement therapy use in women (pre-menopause, post-menopause non-user, post-menopause past user, and post-menopause current user).

c Statistically significant heterogeneity between groups, *p* = 0.046.

AR, age-standardized AR per 100,000 person-years.

No substantial differences were found in the stratified analysis according to age, UV-index, or BMI. When stratified by smoking status, significant associations were found in never-smokers (RR = 1.19; 95% CI 1.08–1.31 in men, and RR = 1.28; 95% CI 1.20–1.38 in women) and past smokers (RR = 1.12; 95% CI 1.03–1.22 in men, and RR = 1.27; 95% CI 1.20–1.35 in women), but not in current smokers (RR = 1.05; 95% CI 0.75–1.46 in men, and RR = 1.10; 95% CI 0.97–1.23 in women). The *p*-value for interaction was 0.046 for men and women combined.

For individual cancer sites (Table 3), a history of NMSC was associated with an increased risk of prostate cancer in men (RR = 1.11; 95% CI 1.02–1.20, *p* = 0.01). The age-standardized AR was 137 per 100,000 person-years. The RR was similar for fatal prostate cancer (RR = 1.17; 95% CI 0.89–1.53). A history of NMSC was also associated with an increased risk of melanoma in men (RR = 1.99; 95% CI 1.63–2.43, *p*<0.0001). The age-standardized AR was 116 per 100,000 person-years. In women, a history of NMSC was associated with an increased risk of breast cancer (RR = 1.19; 95% CI 1.11–1.28, *p*<0.0001; AR = 87 per 100,000 person-years), lung cancer (RR = 1.32; 95% CI 1.14–1.52, *p* = 0.0002; AR = 22 per 100,000 person-years), leukemia (RR = 1.30; 95% CI 1.00–1.69, *p* = 0.05; AR = 7 per 100,000 person-years), kidney cancer (RR = 1.48; 95% CI 1.10–1.99, *p* = 0.01; AR = 8 per 100,000 person-years), and melanoma (RR = 2.58; 95% CI 2.34 –2.98, *p*<0.0001; AR = 79 per 100,000 person-years). After taking into account the multiple comparisons for individual cancers (*n* = 28), the associations with breast cancer, lung cancer in women, and melanoma in both men and women remained significant. We analyzed SCC and BCC history separately for individual cancer sites. We observed different rates for second cancer development according to personal history of SCC and BCC. However, no statistically significant heterogeneity (*p* for heterogeneity ranged from 0.16 to 0.88) was found for any cancer site between BCC and SCC due to the limited power (Table S3).

**Table 3:** Risks of subsequent primary cancers at different sites according to personal history of non-melanoma skin cancer in men and women.

Cancer	Cases		AR	Age-Adjusted RR (95% CI)^a^	Multivariable-Adjusted RR (95% CI)^b^
	No NMSC	NMSC			
**Men (HPFS)**	756,608 person-years	76,888 person-years			
Prostate cancer	4,256	730	137	1.17 (1.08–1.27)	1.11 (1.02–1.20)
Lung cancer	627	97	−6	0.93 (0.74–1.16)	0.99 (0.79–1.24)
Colorectal cancer	889	129	17	1.10 (0.91–1.34)	1.12 (0.92–1.36)
Bladder cancer	513	88	4	1.14 (0.90–1.45)	1.11 (0.87–1.41)
Pancreatic cancer	234	37	3	1.01 (0.70–1.45)	1.06 (0.74–1.53)
Non-Hodgkin lymphoma	312	52	3	1.17 (0.85–1.59)	1.17 (0.86–1.60)
Leukemia	327	51	6	1.08 (0.80–1.48)	1.08 (0.79–1.47)
Kidney cancer	226	31	4	1.09 (0.73–1.61)	1.08 (0.73–1.61)
Melanoma	555	142	116	**2.05 (1.69**–**2.50)**	**1.99 (1.63**–**2.43)** c
Brain tumor	124	15	2	0.90 (0.52–1.57)	0.95 (0.54–1.65)
Oral cancer	92	11	−1	0.95 (0.50–1.82)	0.95 (0.49–1.85)
Multiple myeloma	123	17	1	1.07 (0.62–1.82)	1.09 (0.63–1.87)
Others	790	122	−2	1.10 (0.90–1.35)	1.11 (0.91–1.36)
**Women (NHS)**	1,900,258 person-years	215,920 person-years			
Breast cancer	5,895	928	87	**1.31 (1.22**–**1.41)**	**1.19 (1.11**–**1.28)** c
Endometrial cancer	1,239	168	12	1.13 (0.96–1.34)	1.16 (0.98–1.37)
Ovarian cancer	609	87	5	1.17 (0.93–1.47)	1.13 (0.89–1.42)
Lung cancer	1,298	242	22	**1.35 (1.17**–**1.55)**	**1.32 (1.14**–**1.52)** c
Colorectal cancer	1,439	208	−4	1.03 (0.89–1.19)	1.03 (0.89–1.20)
Bladder cancer	355	67	6	1.29 (0.99–1.68)	1.22 (0.93–1.60)
Pancreatic cancer	254	31	−3	0.89 (0.61–1.31)	0.96 (0.65–1.40)
Non-Hodgkin lymphoma	571	101	6	1.22 (0.98–1.51)	1.15 (0.92–1.43)
Leukemia	363	70	7	1.34 (1.03–1.75)	1.30 (1.00–1.69)
Kidney cancer	269	56	8	1.48 (1.10–1.99)	1.48 (1.10–1.99)
Melanoma	782	288	79	**2.70 (2.34**–**3.11)**	**2.58 (2.34**–**2.98)** c
Brain tumor	124	16	1	1.25 (0.73–2.12)	1.23 (0.72–2.09)
Oral cancer	95	19	6	1.75 (1.06–2.91)	1.64 (0.99–2.73)
Multiple myeloma	161	18	−2	0.80 (0.49–1.31)	0.78 (0.48–1.29)
Others	2,614	490	21	1.08 (0.98–1.20)	1.12 (1.02–1.24)

AR, age-standardized AR per 100,000 person-years.

a RR adjusted for age (continuous variable).

b Multivariate RR adjusted for age (continuous variable), BMI (<21, 21–23, 23–25, 25–27, 27–29, 29–31, >31), physical activity (quintiles), smoking status (never, past 1–14 cigarettes per day, past 15+ cigarettes per day, current 1–14 cigarettes per day, current 15+ cigarettes per day), multi-vitamin use (yes or no), UV-index of residence at birth, age 15, and age 30 (≤5, 6, ≥7), physical examination in the last 2 y (yes or no), and menopause status and hormone replacement therapy use in women (pre-menopause, post-menopause non-user, post-menopause past user, and post-menopause current user). For breast cancer, we additionally included in the multivariate model duration of hormone replacement therapy use (pre-menopause, dubious menopause, post menopause age <48 and never user, post menopause age <48 and past user, post menopause age <48 and current user <5 y, post menopause age <48 and current user ≥5 y, post menopause 48<age<52 and never user, post menopause age 48<age<52 and past user, post menopause age 48<age<52 and current user <5 y, post menopause age 48<age<52 and current user ≥5 y, post menopause age ≥52 and never user, post menopause age ≥52 and past user, post menopause age ≥52 and current user <5 y, post menopause age ≥52 and current user ≥5 y), duration of menopause (continuous variable), alcohol consumption (0 g/d, 0.1–4.9 g/d, 5.0–14.9 g/d, 15.0+ g/d), family history of breast cancer (yes or no), history of benign breast disease (yes or no), height (<160.02 cm, 160.02–162.56 cm, 162.56–167.64 cm, ≥167.64 cm), weight change from age at 18 (loss >4 kg, stable, gain 4–10 kg, gain 10–20 kg, gain 20–40 kg, gain >40 kg), parity and age at first birth (nulliparous, parity 1–2 and age at first birth <25, parity 1–2 and 25<age at first birth <30, parity 1–2 and age at first birth >30, parity 3–4 and age at first birth <25, parity 3–4 and 25<age at first birth <30, parity 3–4 and age at first birth >30, parity >5 and age at first birth <25, parity >5 and 25<age at first birth <30), and age at menarche (≤12 y, 13 y, ≥14 y). For ovarian cancer and endometrial cancer, the multivariate model additionally included duration of hormone replacement therapy use, parity and age at first birth, and age at menarche, categorized as previously. For prostate cancer, the multivariate model additionally included height (<160.02 cm, 160.02–162.56 cm, 162.56–167.64 cm, ≥167.64 cm), BMI at age 21 (<21, 21–23, 23–25, 25–27, 27–29, 29–31, >31), family history of prostate cancer (yes or no), red meat consumption (continuous variable), fruit and vegetable consumption (continuous variable), and history of prostate-specific antigen testing (yes or no, lagged by one period to avoid counting diagnostic prostate-specific antigen tests as screening; collected from 1994 onwards). For melanoma, the multivariate model additionally included childhood reaction to sun (some redness or none, burn, painful burn or blisters), severe sunburns (none, 1–2, 3–5, 6–9, ≥10), moles on the left arm (none, 1–2, 3–5, 6–9, ≥10), hair color (black, dark brown, light brown, blonde, red), family history of melanoma (yes or no), sun exposures at different age intervals (continuous variable), UV index of residence at birth, age 15, and age 30 (≤5, 6, ≥7).

c Statistically significant after correction for multiple comparisons, *p*<0.0018.

## Discussion

To the best of our knowledge, this is the largest prospective study on this topic. In this study, a total of 36,102 cases of NMSC and 29,447 cases of cancers other than NMSC were documented. Among those with a personal history of NMSC, we found a 15% increased risk in men and a 26% increased risk in women of developing a second primary cancer, compared with those who had no such history. A systematic review summarizing previous studies revealed that NMSC is associated with more than 10% increased risk of subsequent primary cancer in registry-based studies and nearly 50% increased risk in cohort studies [16]. Our study has extended previous findings by adding a prospective analysis in two large US cohorts with more than two decades of follow-up. The unique aspects of our study included stratified analyses by other risk factors, disentanglement of surveillance bias, and comprehensive adjustment for potential confounders.

It was speculated that the association between NMSC and subsequent cancer risk may be different among people living in locations with different UV-indexes. Specifically, southern regions have solar UV-B radiation levels that provide sufficient vitamin D to reduce the risk of cancer incidence, and thus inverse associations were more likely to be found. On the contrary, studies that found positive associations were mostly conducted in northern regions where UV-B radiation levels do not provide sufficient vitamin D [17],[18]. In our analysis stratified by UV-index, no substantial differences were found for the associations between NMSC and cancer risk among locations with different UV-indexes. To the extent that some cancers have been suggested to be reduced with higher vitamin D [19], it might be worth noting the fact that while colorectal cancer was not increased in this study, it was not decreased as would be expected if NMSC were a marker for sunlight and thus vitamin D exposure.

Intensified medical surveillance of persons with a history of NMSC is unlikely to explain the increased cancer incidence observed in our study. In our analysis, we adjusted for physical examination in the last 2 y, and the result changed little. After we excluded those diagnosed with other primary cancers within the first 4 y of NMSC diagnosis, the association remained significant. Compared to those with history of invasive SCC or with history of SCC or BCC, individuals with history of SCC in situ were less likely to develop subsequent cancers. These results suggested that the patients who carried NMSC precursors but did not develop skin cancers might be either less genetically susceptible or have lower exposure. In addition, for prostate cancer, the association remained significant for fatal prostate cancer. Furthermore, several cancers that we observed associations with are not ones that would be detected on routine screening. Moreover, at least one study that assessed deaths rather than cancer incidence also found increased cancer mortality in people with history of NMSC [20].

Several studies have observed an increased risk of NMSC after other cancers. In one study, chronic lymphocytic leukemia patients had an increased risk of death due to NMSC (RR = 17.0, 95% CI 14.4–19.8) [21]. In another study, among 14 different sites for first primary malignancies, 11 of these sites including prostate, breast, and leukemia were followed by an increased risk of skin cancer (for SCC, RR of 14.1 for males and 14.6 for females) [22]. However, while treatment of these primary cancers may predispose to subsequent skin cancers, most of the NMSC cases are cured by surgical excision without any systemic chemotherapy, and radiation, and their concomitant side effects, including possible carcinogenicity. In addition, the similarity between the age-adjusted and multivariate-adjusted RRs demonstrated that the observed association between NMSC and subsequent cancers is unlikely to be explained by confounding from smoking, obesity, vitamin use, exercise, or any of the other measured risk factors that we controlled for.

The link between NMSC and risk of other cancers is likely to represent an etiologic association. For melanoma especially, the link may be due to sun exposure. For other cancers, while there are several explanations [23]–[28] of the association between NMSC and the risk of subsequent cancer, studies have found that certain genetic markers underlying skin cancer are also associated with other cancer types [29]. It is biologically plausible that deficiencies of pathways responsible for protecting against cellular transformation in multiple tissues, such as DNA repair or immune responses, may act systemically and play a role in cutaneous and internal carcinogenesis.

Humans have evolved several DNA repair pathways dealing with damage [30]. The nucleotide excision repair (NER) pathway is responsible for the repair of a wide variety of DNA damage that leads to distortion of the DNA helix. Such bulky DNA adducts include UV-induced photoproducts, smoking-related benzo(a)pyrene diolepoxide (BPDE)-DNA adducts, and other DNA damage induced by chemical carcinogens. Reduced capacity of the NER has been shown to confer susceptibility to certain cancers in the general population, including melanoma, BCC, SCC, SCC of head and neck, lung cancer, breast cancer, and bladder cancer [31]–[34]. Personal history of NMSC may be a marker of susceptibility due to reduced DNA repair capacity and it may predict the risk of subsequent cancer development.

The NER activity has been shown to be tissue-specific. For example, relatively low NER efficiency was observed in oral tissues [35]. Both rapidly proliferating tissues (e.g., kidney) and slowly proliferating tissues (e.g., lung) exhibit higher demand for NER capacity upon stimulation to proliferation [36]. The DNA repair system consists of several distinct pathways with many subcomponents, each interacting and overlapping with one another in order to achieve genomic stability and high fidelity. Some tissues, such as breast, lack redundant systems of DNA repair that are present in other tissues [37],[38]. Defects in DNA repair would be expected to have greater impact in such tissues without extensive DNA repair redundancy. In addition, a number of studies have suggested a role of sex hormone (e.g., estrogen and androgen) in the regulation of DNA repair activity in breast and prostate cancers [39]. After correction for multiple comparisons for individual types of cancers, the significant association remained for breast cancer, lung cancer in women, and melanoma in both men and women. Even though the positive associations are biologically plausible, we cannot rule out the possibility of chance findings for each individual cancer site.

In our analysis stratified by smoking status, significant association was found among never and past smokers. Because the NER enzymes recognize bulky DNA adducts including both UV-induced photoproducts and smoking-related BPDE-DNA adducts, the interaction between smoking status and history of NMSC highlights the potential role of the NER pathway in the development of second cancers. However, the effect of inherited insufficient capacity of the NER indicated by history of NMSC is only apparent among non-current smokers and for lung cancer in women. Further mechanistic investigation is warranted.

Sub-optimal immune surveillance could be another common susceptibility factor for both cutaneous and internal cancers. Malignant progression is accompanied by profound immune suppression that interferes with an effective antitumor response and tumor elimination [40]. Impaired immunity has been implicated as a non-site-specific determinant of cancer risk [41]. In addition, UV radiation can also cause immunosuppression. UV exposure adversely affects the skin immune system by diminishing antigen-presenting cell function, inducing immunosuppressive cytokine production, and modulating contact and delayed-type hypersensitivity reactions [42],[43], all of which can reduce the body's surveillance for tumor cells [44],[45]. UV suppresses immune reactions locally, but can also affect the immune system in a systemic fashion when higher UV doses are given [46],[47]. UV radiation affects immune surveillance by modulating the balance between an effective immune response and immune tolerance of an emerging cancer [41]. We did not observe an association between UV-index and the risk of cancer except for skin cancer in our study, which makes this explanation less likely for our findings.

The identification of BCC cases in this study was based on self-report without pathological confirmation. However, the participants in the two cohorts were nurses and health professionals. The validity of their reports was expected to be high, and it has been proven in our validation studies [13],[14]. In addition, previous studies of BCC in the NHS using the self-reported cases identified both constitutional and sun exposure risk factors as expected, such as lighter pigmentation, less childhood and adolescent tanning tendency, higher tendency to sunburn, and tanning salon attendance [48],[49]. We recently confirmed the MC1R gene as the top BCC risk locus using the NHS and HPFS samples [50]. These data together suggest that the bias due to self-report of BCC is likely to be minimal in our study. Moreover, the potential under-report of BCC diagnosis would be expected to bias observed associations toward the null, and such bias would not explain the positive associations that we found.

The strengths of our study included the prospective cohort design and updated assessment of cancer diagnosis and other risk factors every 2 y, more than two decades of follow-up, and a large number of incident skin cancer cases. We had detailed data on related covariates for stratified analyses and comprehensive adjustment for potential confounders. All the participants were health professionals, minimizing potential confounding by educational attainment or differential access to health care. Nevertheless, we cannot completely exclude residual confounding, and our findings may not assign causality. Although the observed significant associations in breast cancer, lung cancer in women, and melanoma in both men and women remained significant after correction for multiple comparisons, we cannot absolutely rule out chance findings for individual cancer sites, and the underlying mechanism for the associations found in specific cancer sites is not entirely clear. In addition, the significant associations for some individual cancers did not meet the adjusted *p*-value threshold because of their limited sample size.

We cannot estimate the recurrence rate of NMSC or subsequent cancer risk among people with multiple NMSC because we only recorded the first report of each type of skin cancer in both cohorts. We do not have data for non-whites in this study, and our results cannot be generalized to non-whites owing to the dramatic difference in skin cancer incidence among different races. In summary, we observed a modestly increased risk of other cancers among individuals with a history of NMSC. Because our study was observational, these results should be interpreted cautiously and are insufficient evidence to alter current clinical recommendations. Nevertheless, these data support a need for continued investigation of the potential mechanisms underlying this relationship.

## Supporting Information

Table S1 Overall analysis of risks of total subsequent primary cancers according to personal history of SCC and BCC.
(DOCX)

Table S2 Overall analysis of risks of total subsequent primary cancers according to personal history of SCC in situ.
(DOCX)

Table S3 Risks of subsequent primary cancers at different sites according to personal history of SCC and BCC.
(DOCX)

## Abbreviations

AR: absolute risk
BCC: basal cell carcinoma
BMI: body mass index
HPFS: Health Professionals Follow-up Study
MV: multivariate
NER: nucleotide excision repair
NHS: Nurses' Health Study
NMSC: non-melanoma skin cancer
RR: relative risk
SCC: squamous cell carcinoma
UV: ultraviolet
