# Supplementary material for: Risk of a Second Primary Cancer after Non-melanoma Skin Cancer in White Men and Women: A Prospective Cohort Study
Source: PLoS Med. 2013 Apr 23;10(4):e1001433. doi: 10.1371/journal.pmed.1001433 (PMC3635863; doi:10.1371/journal.pmed.1001433)
Supplement: Table S1 — Overall analysis of risks of total subsequent primary cancers according to personal history of SCC and BCC. (DOCX) [file pmed.1001433.s001.docx]

**Table S1: Overall analysis of risks of total subsequent primary cancers according to personal history of SCC and BCC**

| **Group** | **Men (HPFS)** | | | | **Women (NHS)** | | | |
| --- | --- | --- | --- | --- | --- | --- | --- | --- |
|  | **Cases / person-years** | | **Age-adjusted**  **RR (95% CI)^a^** | **Multivariate-adjusted RR (95% CI)^b^** | **Cases / person-years** | | **Age-adjusted**  **RR (95% CI)^a^** | **Multivariate-adjusted RR (95% CI)^b^** |
|  | **BCC (ref)** | **SCC** |  |  | **BCC (ref)** | **SCC** |  |  |
| Overall | 1,337 / 67,446 | 185 / 9,442 | 0.94 (0.80, 1.11) | 0.93 (0.79, 1.09) | 2,507 / 195,512 | 282 / 20,408 | 1.01 (0.89, 1.14) | 1.00 (0.88, 1.14) |
| Overall excluding melanoma | 1,219 / 66,598 | 161 / 9,318 | 0.90 (0.76, 1.07) | 0.89 (0.75, 1.06) | 2,260 / 193,187 | 241 / 20,162 | 0.97 (0.84, 1.11) | 0.96 (0.84, 1.10) |

^a^: Relative risk adjusted for age (continuous variable).

^b^: Multivariate relative risk adjusted for age (continuous variable), BMI (<21, 21-23, 23-25, 25-27, 27-29, 29-31, >31), physical activity (quintiles), smoking status (never, past 1-14 cigarettes per day, past 15+ cigarettes per day, current 1-14 cigarettes per day, current 15+ cigarettes per day), multi-vitamin use (yes or no), UV-index of residence at birth, age 15, and age 30 (≤ 5, 6, ≥ 7), physical examination in the last two years (yes or no) , and menopause status and hormone replacement therapy use in women (pre-menopause, post-menopause non-user, post-menopause past user, and post-menopause current user).
