# Supplementary material for: Risk of a Second Primary Cancer after Non-melanoma Skin Cancer in White Men and Women: A Prospective Cohort Study
Source: PLoS Med. 2013 Apr 23;10(4):e1001433. doi: 10.1371/journal.pmed.1001433 (PMC3635863; doi:10.1371/journal.pmed.1001433)
Supplement: Table S2 — Overall analysis of risks of total subsequent primary cancers according to personal history of SCC in situ. (DOCX) [file pmed.1001433.s002.docx]

**Table S2: Overall analysis of risks of total subsequent primary cancers according to personal history of SCC in situ**

| **Group** | **Men (HPFS)** | | | | | | **Women (NHS)** | | | | | | |
| --- | --- | --- | --- | --- | --- | --- | --- | --- | --- | --- | --- | --- | --- |
|  | **Cases** | **person-years** | **Age-adjusted RR (95% CI)^a^** | **Multivariate-adjusted**  **RR (95% CI)^b^** | | | **Cases** | **person-years** | | **Age-adjusted RR (95% CI)^a^** | | **Multivariate-adjusted**  **RR (95% CI)^b^** | |
| Overall | | | | | | | | | | | | | |
| SCC invasive | 185 | 9,442 | 1.00 | 1.00 | | | 282 | 20,408 | | 1.00 | | 1.00 | |
| SCC in situ | 25 | 1,682 | 0.69 (0.38, 1.23) | 0.81 (0.43, 1.50) | | | 34 | 3,978 | | 0.59 (0.40, 0.89) | | 0.59 (0.39, 0.90) | |
| SCC or BCC | 1,522 | 76,888 | 1.00 | 1.00 | | | 2,789 | 215,920 | | 1.00 | | 1.00 | |
| SCC in situ | 25 | 1682 | 0.76 (0.50, 1.15) | 0.74 (0.48, 1.13) | | | 34 | 3,978 | | 0.63 (0.45, 0.89) | | 0.63 (0.44, 0.88) | |
| Overall excluding melanoma | | | | | | | | | | | | | |
| SCC invasive | 161 | 9,318 | 1.00 | | 1.00 | 241 | | | 20,162 | | 1.00 | | 1.00 |
| SCC in situ | 23 | 1,676 | 0.70 (0.37, 1.31) | | 0.91 (0.47, 1.77) | 30 | | | 3,923 | | 0.61 (0.39, 0.94) | | 0.61 (0.39, 0.95) |
| SCC or BCC | 1,380 | 759,16 | 1.00 | | 1.00 | 2,501 | | | 213,349 | | 1.00 | | 1.00 |
| SCC in situ | 23 | 1,676 | 0.75 (0.49, 1.17) | | 0.73 (0.47, 1.14) | 30 | | | 3,923 | | 0.64 (0.44, 0.91) | | 0.66 (0.44, 0.92) |

^a^: Relative risk adjusted for age (continuous variable).

^b^: Multivariate relative risk adjusted for age (continuous variable), BMI (<21, 21-23, 23-25, 25-27, 27-29, 29-31, >31), physical activity (quintiles), smoking status (never, past 1-14 cigarettes per day, past 15+ cigarettes per day, current 1-14 cigarettes per day, current 15+ cigarettes per day), multi-vitamin use (yes or no), UV-index of residence at birth, age 15, and age 30 (≤ 5, 6, ≥ 7), physical examination in the last two years (yes or no) , and menopause status and hormone replacement therapy use in women (pre-menopause, post-menopause non-user, post-menopause past user, and post-menopause current user).
