# Supplementary material for: Risk of a Second Primary Cancer after Non-melanoma Skin Cancer in White Men and Women: A Prospective Cohort Study
Source: PLoS Med. 2013 Apr 23;10(4):e1001433. doi: 10.1371/journal.pmed.1001433 (PMC3635863; doi:10.1371/journal.pmed.1001433)
Supplement: Table S3 — Risks of subsequent primary cancers at different sites according to personal history of SCC and BCC. (DOCX) [file pmed.1001433.s003.docx]

**Table S3: Risks of subsequent primary cancers at different sites according to personal history of SCC and BCC**

| **Group** | **Men (HPFS)** | | | | **Women (NHS)** | | | |
| --- | --- | --- | --- | --- | --- | --- | --- | --- |
|  | **Cases / person-years** | | **Age-adjusted**  **RR (95% CI)^a^** | **Multivariate-adjusted RR (95% CI)^b^** | **Cases / person-years** | | **Age-adjusted**  **RR (95% CI)^a^** | **Multivariate-adjusted RR (95% CI)^b^** |
|  | **No NMSC** | **NMSC** |  |  | **No NMSC** | **NMSC** |  |  |
| Prostate cancer after SCC | 4,903 / 824,054 | 83 / 9,442 | 1.00 (0.80, 1.25) | 0.96 (0.77, 1.20) |  |  |  |  |
| Prostate cancer after BCC | 4,339 / 766,050 | 647 / 67,446 | 1.19 (1.09, 1.30) | 1.13 (1.04, 1.23) |  |  |  |  |
| Breast cancer after SCC |  |  |  |  | 6,724 / 2,095,770 | 99 / 20,408 | 1.45 (1.19, 1.78) | 1.34 (1.10, 1.64) |
| Breast cancer after BCC |  |  |  |  | 5,994 / 1,920,665 | 829 / 195,512 | 1.28 (1.19, 1.38) | 1.17 (1.08, 1.26) |
| Endometrial cancer after SCC |  |  |  |  | 1,392 / 2,095,770 | 15 / 20,408 | 1.15 (0.69, 1.92) | 1.20 (0.72, 2.01) |
| Endometrial cancer after BCC |  |  |  |  | 1,254 / 1,920,665 | 153 / 195,512 | 1.13 (0.95, 1.34) | 1.15 (0.97, 1.36) |
| Ovarian cancer after SCC |  |  |  |  | 690 / 2,095,770 | 6 / 20,408 | 0.90 (0.40, 2.01) | 0.85 (0.38, 1.91) |
| Ovarian cancer after BCC |  |  |  |  | 615 / 1,920,665 | 81 / 195,512 | 1.19 (0.94, 1.51) | 1.15 (0.91, 1.46) |
| Lung cancer after SCC | 715 / 824,054 | 9 / 9,442 | 0.58 (0.30, 1.13) | 0.58 (0.30, 1.13) | 1,518 / 2,095,770 | 22 / 20,408 | 1.23 (0.80, 1.88) | 1.09 (0.71, 1.68) |
| Lung cancer after BCC | 636 / 766,050 | 88 / 67,446 | 1.01 (0.80, 1.27) | 1.09 (0.86, 1.38) | 1,320 / 1,920,665 | 220 / 195,512 | 1.35 (1.17, 1.56) | 1.33 (1.14, 1.53) |
| Colorectal cancer after SCC | 1,004 / 824,054 | 14 / 9,442 | 0.84 (0.49, 1.44) | 0.84 (0.49, 1.44) | 1,627 / 2,095,770 | 20 / 20,408 | 1.01 (0.65, 1.58) | 0.99 (0.63, 1.54) |
| Colorectal cancer after BCC | 903 / 766,050 | 115 / 67,446 | 1.16 (0.95, 1.42) | 1.17 (0.96, 1.44) | 1,459 / 1,920,665 | 188 / 195,512 | 1.03 (0.88, 1.20) | 1.03 (0.89, 1.21) |
| Bladder cancer after SCC | 586 / 824,054 | 15 / 9,442 | 1.34 (0.79, 2.27) | 1.24 (0.73, 2.11) | 422 / 2,095,770 | 0 / 20,408 | -- | -- |
| Bladder cancer after BCC | 582 / 766,050 | 73 / 67,446 | 1.07 (0.83, 1.39) | 1.05 (0.81, 1.37) | 355 / 1,920,665 | 67 / 195,512 | 1.45 (1.11, 1.89) | 1.38 (1.05, 1.80) |
| Pancreatic cancer after SCC | 266 / 824,054 | 5 / 9,442 | 0.97 (0.40, 2.39) | 0.98 (0.40, 2.42) | 283 / 2,095,770 | 2 / 20,408 | 0.66 (0.16, 2.67) | 0.64 (0.16, 2.61) |
| Pancreatic cancer after BCC | 239 / 766,050 | 32 / 67,446 | 1.03 (0.70, 1.51) | 1.09 (0.74, 1.59) | 256 / 1,920,665 | 29 / 195,512 | 0.92 (0.62, 1.36) | 1.00 (0.47, 1.48) |
| Non-Hodgkin lymphoma after SCC | 356 / 824,054 | 8 / 9,442 | 1.32 (0.65, 2.71) | 1.35 (0.66, 2.77) | 659 / 2,095,770 | 13 / 20,408 | 1.48 (0.85, 2.57) | 1.41 (0.81, 2.46) |
| Non-Hodgkin lymphoma after BCC | 320 / 766,050 | 44 / 67,446 | 1.12 (0.81, 1.57) | 1.13 (0.81, 1.57) | 584 / 1,920,665 | 88 / 195,512 | 1.17 (0.93, 1.47) | 1.11 (0.88, 1.39) |
| Leukemia after SCC | 372 / 824,054 | 6 / 9,442 | 0.88 (0.38, 2.03) | 0.86 (0.37, 2.00) | 424 / 2,095,770 | 9 / 20,408 | 1.64 (0.84, 3.20) | 1.58 (0.81, 3.09) |
| Leukemia after BCC | 333 / 766,050 | 45 / 67,446 | 1.13 (0.81, 1.55) | 1.12 (0.81, 1.55) | 372 / 1,920,665 | 61 / 195,512 | 1.29 (0.98, 1.69) | 1.24 (0.94, 1.64) |
| Kidney cancer after SCC | 253 / 824,054 | 4 / 9,442 | 1.21 (0.44, 3.32) | 1.23 (0.45, 3.38) | 325 / 2,095,770 | 0 / 20,408 | -- | -- |
| Kidney cancer after BCC | 210 / 766,050 | 47 / 67,446 | 1.06 (0.70, 1.62) | 1.06 (0.69, 1.61) | 269 / 1,920,665 | 56 / 195,512 | 1.67 (1.24, 2.34) | 1.67 (1.24, 2.34) |
| Melanoma after SCC | 673 / 824,054 | 24 / 9,442 | 2.26 (1.48, 3.44) | 2.19 (1.43, 3.34) | 1029 / 2,095,770 | 41 / 20,408 | 2.90 (2.11, 3.99) | 2.73 (1.98, 3.75) |
| Melanoma after BCC | 579 / 766,050 | 118 / 67,446 | 1.89 (1.53, 2.33) | 1.83 (1.48, 2.26) | 823 / 1,920,665 | 247 / 195,512 | 2.46 (2.12, 2.85) | 2.36 (2.03, 2.73) |
| Brain tumor after SCC | 138 / 824,054 | 1 / 9,442 | 0.55 (0.08, 4.00) | 0.56 (0.08, 4.06) | 139 / 2,095,770 | 1 / 20,408 | 0.97 (0.13, 6.99) | 0.93 (0.13, 6.74) |
| Brain tumor after BCC | 125 / 766,050 | 14 / 67,446 | 0.95 (0.54, 1.68) | 1.00 (0.56, 1.77) | 125 / 1,920,665 | 15 / 195,512 | 1.27 (0.73, 2.18) | 1.25 (0.72, 2.16) |
| Oral cancer after SCC | 101 / 824,054 | 2 / 9,442 | 1.28 (0.30, 5.37) | 1.25 (0.29, 5.35) | 112 / 2,095,770 | 2 / 20,408 | 1.93 (0.47, 7.93) | 1.78 (0.43, 7.33) |
| Oral cancer after BCC | 94 / 766,050 | 9 / 67,446 | 0.90 (0.44, 1.81) | 0.92 (0.45, 1.89) | 97 / 1,920,665 | 17 / 195,512 | 1.69 (1.00, 2.86) | 1.59 (0.94, 2.70) |
| Multiple myeloma after SCC | 138 / 824,054 | 2 / 9,442 | 0.76 (0.18, 3.13) | 0.79 (0.19, 3.29) | 179 / 2,095,770 | 0 / 20,408 | -- | -- |
| Multiple myeloma after BCC | 125 / 766,050 | 15 / 67,446 | 1.07 (0.60, 1.90) | 1.08 (0.60, 1.93) | 161 / 1,920,665 | 18 / 195,512 | 0.91 (0.55, 1.48) | 0.88 (0.54, 1.45) |
| Other cancer after SCC | 900 / 824,054 | 12 / 9,442 | 0.77 (0.43, 1.38) | 0.78 (0.44, 1.39) | 3,052 / 2,095,770 | 52 / 20,408 | 1.03 (0.78, 1.35) | 1.05 (0.80, 1.38) |
| Other cancer after BCC | 802 / 766,050 | 110 / 67,446 | 1.14 (0.92, 1.41) | 1.15 (0.93, 1.42) | 2,666 / 1,920,665 | 438 / 195,512 | 1.09 (0.98, 1.21) | 1.13 (1.02, 1.25) |

^a^: Relative risk adjusted for age (continuous variable).

^b^: Multivariate relative risk adjusted for age (continuous variable), BMI (<21, 21-23, 23-25, 25-27, 27-29, 29-31, >31), physical activity (quintiles), smoking status (never, past 1-14 cigarettes per day, past 15+ cigarettes per day, current 1-14 cigarettes per day, current 15+ cigarettes per day), multi-vitamin use (yes or no), UV-index of residence at birth, age 15, and age 30 (≤ 5, 6, ≥ 7), physical examination in the last two years (yes or no) , and menopause status and hormone replacement therapy use in women (pre-menopause, post-menopause non-user, post-menopause past user, and post-menopause current user). For breast cancer, we additionally included in the multivariate model duration of hormone replacement therapy use (pre-menopause, dubious menopause, post menopause age <48 and never user, post menopause age <48 and past user, post menopause age <48 and current user <5 years, post menopause age <48 and current user ≥5 years, post menopause 48<age<52 and never user, post menopause age 48<age<52 and past user, post menopause age 48<age<52 and current user <5 years, post menopause age 48<age<52 and current user ≥5 years, post menopause age ≥52 and never user, post menopause age ≥52 and past user, post menopause age ≥52 and current user <5 years, post menopause age ≥52 and current user ≥5 years), duration of menopause (continuous variable), alcohol consumption (0 g/d, 0.1-4.9 g/d, 5.0-14.9 g/d, 15.0+ g/d), family history of breast cancer (yes or no), history of benign breast disease (yes or no), height (<63 inches, 63-63.9 inches, 64-65.9 inches, ≥66 inches), weight change from age at 18 (loss >4 kg, stable, gain 4-10 kg, gain 10-20 kg, gain 20-40 kg, gain >40 kg), parity and age at first birth (nulliparous, parity 1-2 and age at first birth <25, parity 1-2 and 25 < age at first birth <30, parity 1-2 and age at first birth >30, parity 3-4 and age at first birth <25, parity 3-4 and 25 < age at first birth <30, parity 3-4 and age at first birth >30, parity >5 and age at first birth <25, parity >5 and 25 < age at first birth <30), and age at menarche (≤ 12 years, 13 years, ≥14 years). For ovarian cancer and endometrial cancer, the multivariate model additionally included duration of hormone replacement therapy use, parity and age at first birth, and age at menarche, categorized as previous. For prostate cancer, the multivariate model additionally included height (<63 inches, 63-63.9 inches, 64-65.9 inches, ≥66 inches), BMI at age 21 (<21, 21-23, 23-25, 25-27, 27-29, 29-31, >31), family history of prostate cancer (yes or no), red meat consumption (continuous variable), fruit and vegetable consumption (continuous variable) , and history of prostate-specific antigen testing (yes or no, lagged by one period to avoid counting diagnostic prostate-specific antigen tests as screening; collected from 1994 onwards). For melanoma, the multivariate model additionally included childhood reaction to sun (some redness or none, burn, painful burn or blisters), severe sunburns (none, 1-2, 3-5, 6-9, ≥10), moles on the left arm (none, 1-2, 3-5, 6-9, ≥10), hair color (black, dark brown, light brown, blonde, red), family history of melanoma (yes or no), sun exposures at different age intervals (continuous variable), UV index of residence at birth, age 15, and age 30 (≤ 5, 6, ≥7).
